# The Two Challenges in Management of Gastric Glomus Tumors

**DOI:** 10.7759/cureus.9251

**Published:** 2020-07-17

**Authors:** Sheena Mago, Anusha Pasumarthi, David R Miller, Rayan Saade, Micheal Tadros

**Affiliations:** 1 Internal Medicine, University of Connecticut Health Center, Farmington, USA; 2 Gastroenterology, Albany Medical Center, Albany, USA; 3 Pathology, Albany Medical Center, Albany, USA; 4 Gastroenterology and Hepatology, Albany Medical Center, Albany, USA

**Keywords:** gastric glomus tumors, ggt, histology, gastrointestinal stromal tumors, gist

## Abstract

Gastric glomus tumors (GGTs) are rare gastrointestinal lesions originating from the neuromuscular arterial canal or vascular lumen which share many overlapping features with other stromal lesions. Despite most cases of GGTs being benign, there is a lack of reliable histological features predictive of tumor behavior. We present a case of a 42-year-old male who was determined to have a GGT via histological diagnosis and underwent surgical wedge resection. This case highlights the importance of establishing an accurate diagnosis and the various factors that must be taken into consideration to best determine malignant potential and management options.

## Introduction

Gastric glomus tumors (GGTs) are rare gastrointestinal lesions originating from the neuromuscular arterial canal or vascular lumen [1]. These lesions are more commonly present in the peripheral soft tissue, however, can seldom be found in the gastrointestinal tract [1]. Since 1951, a few cases have been published in the literature; however, there is still a limited fund of knowledge in the diagnosis and management of this disease [1,2]. Although these mesenchymal neoplasms have a distinct gross appearance and microscopic morphology, GGTs share many overlapping features with other more insidious stromal lesions such as gastrointestinal stromal tumors (GISTs) and carcinoid tumors [1,3]. Despite most cases of GGTs being benign, there is a lack of reliable histological features that are predictive of tumor behavior - making the assessment of prognostication challenging [4,5]. This case highlights the importance of establishing an accurate diagnosis, the various factors that must be taken into consideration to best determine malignant potential, and the importance of determining an appropriate management course with shared decision making.

## Case presentation

A 42-year-old male with hypertension and gastroesophageal reflux disease presented with a three-month history of intermittent sharp left upper quadrant pain and multiple episodes of non-bloody, non-bilious emesis. He had no fevers, chills, hematemesis, unintentional weight changes, jaundice, or changes in stool color and consistency. He was a non-smoker and did not have a history of illicit drug or alcohol use, recent travel history, or non-steroidal anti-inflammatory drug use. His only medications were amlodipine 5 mg and omeprazole 20 mg daily. He also had no previous abdominal surgeries, and there was no significant personal or family history of malignancy.

**Figure 1:**
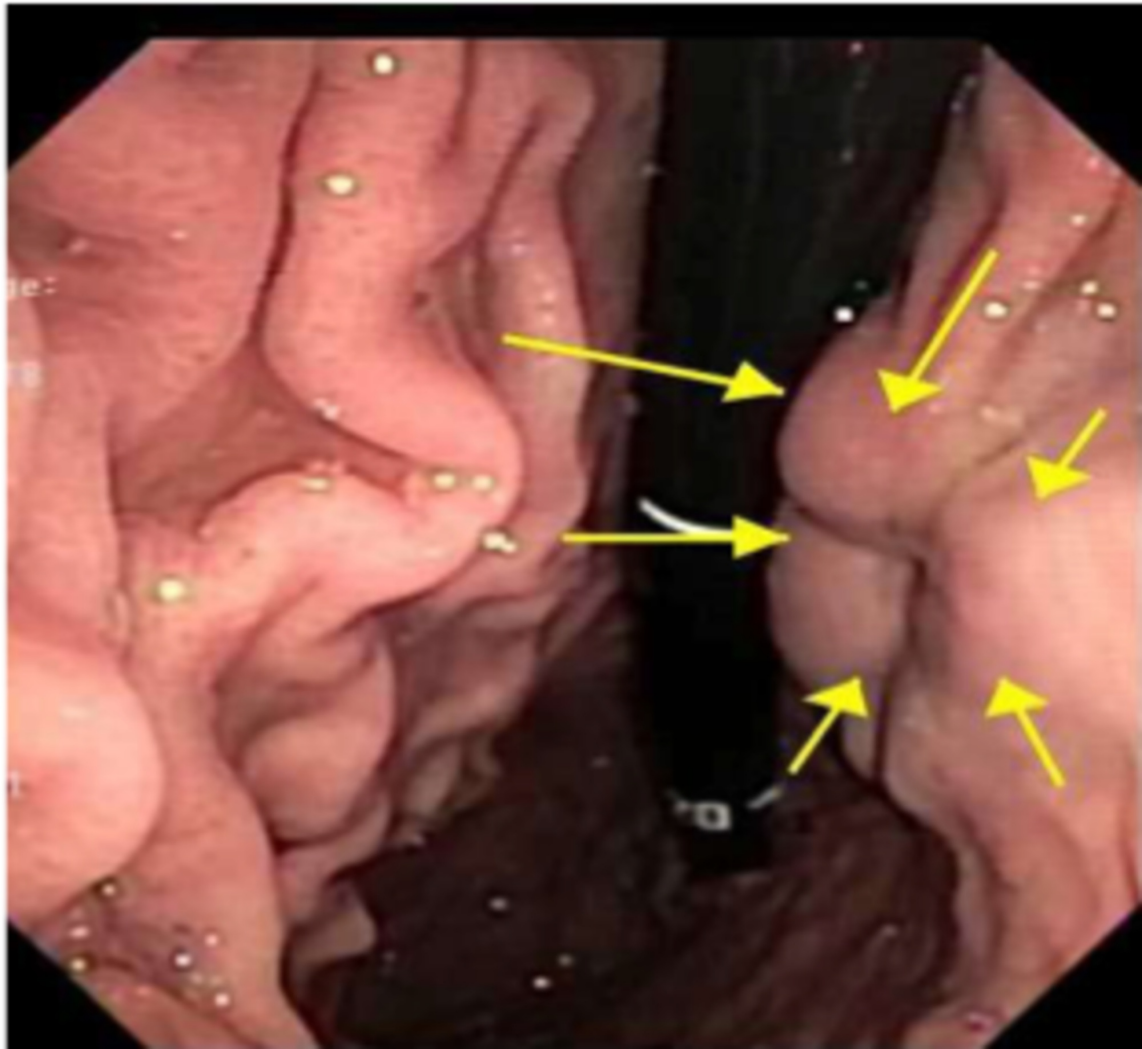
EGD showing a large, submucosal mass with no bleeding and no stigmata of recent bleeding was found at the incisura (yellow arrows) EGD: esophagogastroduodenoscopy.

**Figure 2:**
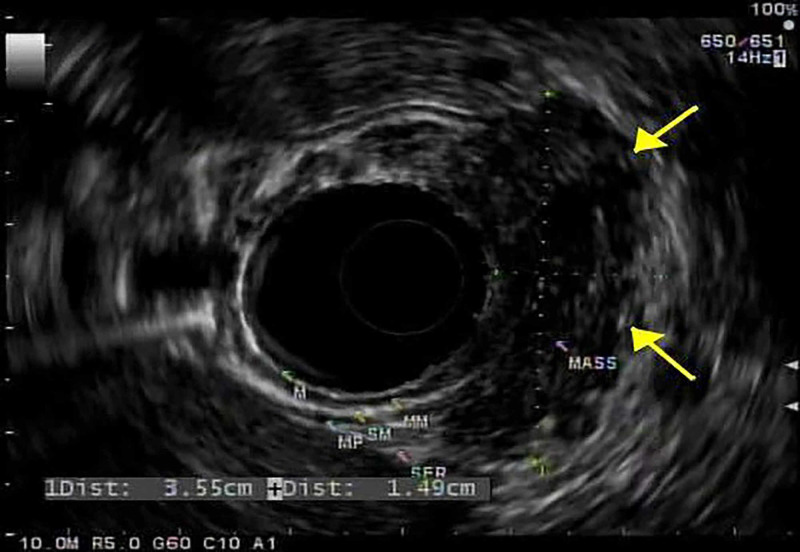
EUS showing a hypoechoic mass in the stomach incisura angularis (yellow arrows) EUS: endoscopic ultrasonography.

He was noted to be afebrile, hemodynamically stable, and had a soft, non-distended abdomen with minimal tenderness of the left upper quadrant with normal bowel sounds, no guarding, or rebound tenderness. Examination was otherwise unremarkable. Laboratory tests were notable only for mild iron deficiency anemia. He had no leukocytosis, transaminitis, hyperbilirubinemia, thrombocytopenia, azotemia, or elevated troponin. Contrast‐enhanced computed tomography (CT) of the abdomen showed a 3.2 x 2.7 x 3.1 cm^3^ soft tissue mass with central calcification and vascularity along the lesser curvature of the stomach. He was admitted to the hospital and underwent esophagogastroduodenoscopy (EGD) which demonstrated a 3 cm submucosal ulcerated mass at the incisura (Figure 1). For further delineation of this mass, an endoscopic ultrasonography (EUS) was conducted, which showed a well-defined hypoechoic lesion arising from the muscularis propria (EUS layer 4) with a maximum diameter of 3.3 cm (Figure 2).

**Figure 3:**
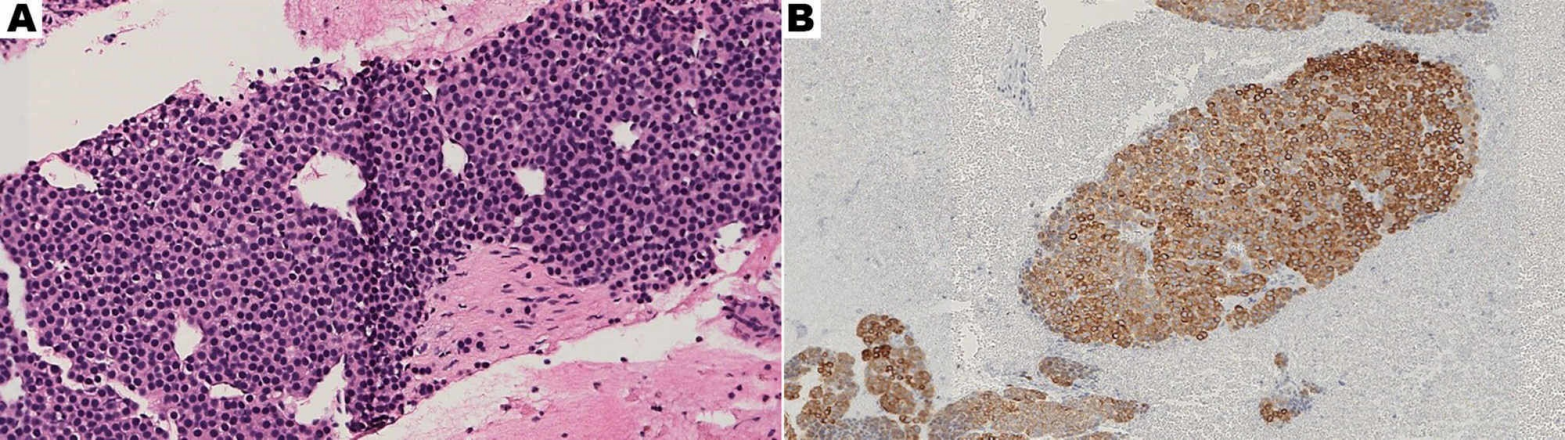
(A) Hematoxylin and eosin (H&E) staining of FNA (30x magnification) and (B) SMA stain on FNA specimen which is characteristically positive in glomus tumors (16x magnification) FNA: fine-needle aspiration, SMA: smooth muscle actin.

Further investigation with fine-needle aspiration (FNA) of the lesion demonstrated small, round, and uniform cells intermixed with capillary-sized vessels (Figure 3A). Immunohistochemistry was performed and revealed positivity for smooth muscle actin (SMA) and synaptophysin, along with weak staining for placental alkaline phosphatase (PLAP) suggestive of a glomus tumor (Figure 3B). The tumor cells lacked markers more indicative of an epithelioid GIST including: CD117, CD34, Desmin, S-100, and DOG1 [6,7]. The pathology also revealed in-tumor-calcification, mild pleomorphism, and sparse Ki67 staining; but no evidence of mitotic activity was noted.

Although EUS and FNA showed small, benign cells, there were concerning risk factors for malignancy including, the patient’s young age, symptomatic presentation, ulcerated lesion appearance, size of the lesion, and neovascularity [8]. After discussing surveillance versus endoscopic and surgical resection options with the patient, a surgical wedge resection of the tumor was performed for definitive management [9,10]. Ultimately, the pathology of the excision specimen was found to be consistent with a benign GGT. On six months follow up, the patient recovered well and symptoms resolved.

## Discussion

The presence of GGTs accounts for only 1% of all gastric soft tissue tumors with a majority being benign and only a few malignant cases of unknown percentage were documented in the literature [11]. Certain tumors, including glomus and GISTs, specifically have variable and non-specific characteristics that make differentiating the two entities a challenging task (Table 1) [12]. Given the different surveillance and treatment guidelines, it is essential to make an accurate diagnosis.

**Table 1:** Characteristics of gastrointestinal subepithelial lesions EUS: endoscopic ultrasonography, EGD: esophagogastroduodenoscopy, SMA: smooth muscle actin, GIST: gastrointestinal stromal tumor, PAS: periodic acid-Schiff.

Type of Subepithelial Lesion	High Propensity for Malignancy	Layer of Origin	Immunohistochemistry	Commonly Found Location	EUS or EGD Findings	Gross Appearance of Biopsy	Histology	When to Consider Surgical Resection
GIST	Yes	4th	Positive: CD117, DOG1	Stomach	Variable; hypoechoic and homogenous lesions or heterogeneous with anechoic spaces	Smooth mucosa with nodules	Variable histological pattern. Two basic cell types are seen: spindle cells and epithelioid cells.	Symptomatic, regional adenopathy, >2 cm, originating in the small bowel
Leiomyoma	No	2nd, 3rd, or 4th	Positive: desmin, α-SMA protein / negative: CD117, CD34, s100	Esophagus	Homogenous hypoechoic, well-defined outline, with internal hyperechoic foci	Well-circumscribed tumors with a white-gray and whorled gross section	Low or moderate cellularity lesions with bland, spindle-shaped cells with cigar-shaped nuclei, mild cytological atypia, and mitoses.	Symptomatic
Carcinoid tumors	Yes	2nd or 3rd	Positive: chromogranin	Small intestine	Homogeneous, hypoechoic or isoechoic, round with smooth margins	Small round, smooth yellow irregularly shaped nodules with depression, ulceration, or fibrosis	Round cells with central round nuclei in a trabecular or rosette pattern with eosinophilic granular cytoplasm.	Symptomatic, regional adenopathy, >2 cm, originating in the small bowel
Glomus tumor	NO	3rd and 4th	Positive: actin, vimentin / negative: CD117, chromogranin A, carcinoembryonic antigen, neuron-specific enolase	Skin, (within gastrointestinal tract: stomach)	Variable echogenicity, internal hyperechoic foci and +Doppler	Red-blue nodules	Small uniformly rounded cells in the walls of dilated vascular spaces with small uniform nuclei surrounded with hyperplastic smooth-muscle cells	Symptomatic, regional adenopathy, >2 cm, originating in the small bowel
Lipomas	No	3rd	N/A	Colon and stomach	Hyperechoic, EGD demonstrates yellow hue, soft, and deformable (classic ‘pillow’ sign)	Compressible, round, well-circumscribed nodule with a homogeneous yellow cross-section	Mature adipose tissue can have inflammation, fat necrosis, fatty cysts, or foamy macrophages if ulcerated	Symptomatic
Granular cell tumors	No	2nd or 3rd	Positive: S100 protein / negative: desmin, actin, CD34, c-kit	Esophagus	Homogeneous, hypoechoic, smooth margins	Firm, pale-yellow nodules, less than 2 cm in diameter	Large oval cells, small nucleus, with basophilic cytoplasm PAS-positive and granular	If concerning risk-features on surveillance endoscopies
Varices	No	3rd	N/A	Esophagus and stomach	Homogeneous, hypoechoic, smooth margins	N/A	N/A	N/A

This case demonstrates that GGTs pose two challenges: (1) differentiation given the significant overlap with more common stromal and mesenchymal tumors and (2) prediction of tumor behavior given the lack of reliable histological features. It is imperative to know the immunohistochemical staining associated with glomus tumors (positive for SMA and PLAP) in order to differentiate them from other submucosal tumors due to their overlapping histological appearance [6,7]. Despite most GGTs being benign, clinicians should consider factors such as patients age, symptoms, tumor size, neovascularity, gross appearance, degree of pleomorphism, Ki67 staining, and mitotic activity to determine malignant potential [12-14]. As seen in this case, there were several concerning factors that were considered for proper risk stratification and prognostication and so adequate shared decision-making for a treatment plan could be conducted between the physician and patient. Due to the rarity of glomus tumors and limited published knowledge, physician discretion is paramount in determining the course of treatment ranging from surveillance to endoscopic or surgical intervention [9,10].

**Figure 4:**
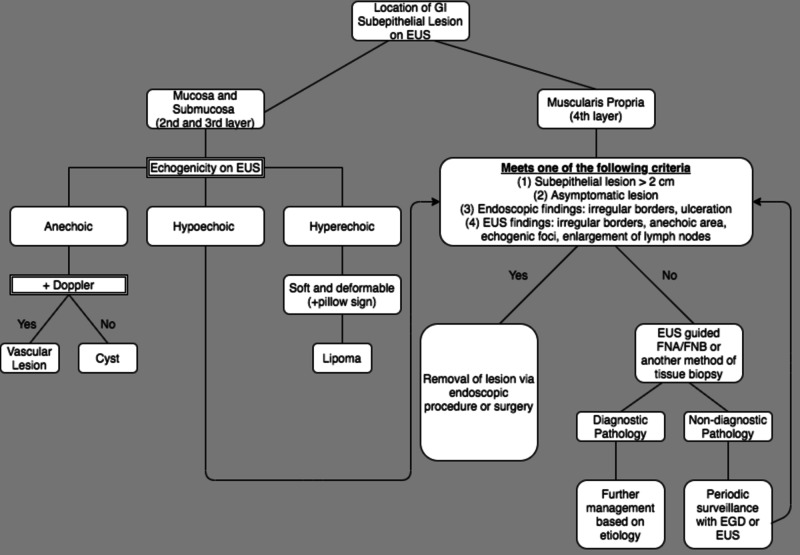
Management for gastrointestinal subepithelial lesions GI: gastrointestinal, EUS: endoscopic ultrasonography, EGD: esophagogastroduodenoscopy, FNA: fine-needle aspiration, FNB: fine-needle biopsy.

The management of subepithelial lesions is largely based on the penetrating mucosal levels (Figure 4). For lesions invading the muscularis propria (layer 4) with concerning risk factors for malignancy, it is recommended that surgical or endoscopic removal of the lesion be conducted. Other concerning factors suggestive of malignancy include: symptomatic lesions (abdominal pain, obstruction, bleeding, and dysphagia), lesion greater than 2 cm, endoscopic evidence of irregular borders or ulceration, or EUS demonstrating lymphadenopathy, anechoic areas of necrosis, or echogenic foci of bleeding/vascularity [7,15]. Given that “bite-on-bite” punch biopsies have low diagnostic yield, it is recommended that an EUS-guided biopsy of the lesion be obtained via FNA or fine-needle biopsy (FNB). If histology is indicative of a benign lesion, regular surveillance endoscopies are recommended for the assessment of evolving risk factors that may necessitate resection. As a less invasive alternative to surgery, these endoscopic methods have the potential to increase patient compliance due to decreased risk of procedural complications [12].

## Conclusions

GGTs are rare gastrointestinal lesions that are predominately benign, however, share many significant overlapping features with other common stromal and mesenchymal lesions which cause challenges in prediction of tumor behavior given the lack of reliable histological features. Due to the rarity of glomus tumors and limited published knowledge, this case reflects the importance of establishing an accurate diagnosis. Factors such as age, symptoms, tumor size, neovascularity, gross appearance, degree of pleomorphism, Ki67 staining, and mitotic activity must be taken into consideration to best determine malignant potential and management options ranging from surveillance to endoscopic or surgical intervention.

## References

[1] Fang HQ, Yang J, Zhang FF, Cui Y, Han AJ (2010). Clinicopathological features of gastric glomus tumor. World J Gastroenterol.

[2] Kay S, Callahan WP, Murray MR, Randall HT, Stout AP (1951). Glomus tumors of the stomach. Cancer.

[3] Wiech T, Walch A, Werner M (2005). Histopathological classification of nonneoplastic and neoplastic gastrointestinal submucosal lesions. Endoscopy.

[4] Lorber J, Kalish J, Farraye FA, Cerda S, Babineau TJ (2005). Glomus tumor of the gastric antrum: case report. Curr Surg.

[5] Agawa H, Matsushita M, Nishio A, Takakuwa H (2002). Gastric glomus tumor. Gastrointest Endosc.

[6] Porter PL, Bigler SA, McNutt M, Gown AM (1991). The immunophenotype of hemangiopericytomas and glomus tumors, with special reference to muscle protein expression: an immunohistochemical study and review of the literature. Mod Pathol.

[7] Cho JW, Korean ESD Study Group (2016). Current guidelines in the management of upper gastrointestinal subepithelial tumors. Clin Endosc.

[8] Pidhorecky I, Cheney RT, Kraybill WG, Gibbs JF (2000). Gastrointestinal stromal tumors: current diagnosis, biologic behavior, and management. Ann Surg Oncol.

[9] Xu M, Jiang XM, He YL, Zhang YL, Xu MD, Yao LQ (2011). Glomus tumor of the stomach: a case treated by endoscopic submucosal dissection. Clin Res Hepatol Gastroenterol.

[10] Miettinen M, Paal E, Lasota J, Sobin LH (2002). Gastrointestinal glomus tumors: a clinicopathologic, immunohistochemical, and molecular genetic study of 32 cases. Am J Surg Pathol.

[11] Folpe AL, Fanburg-Smith JC, Miettinen M, Weiss SW (2001). Atypical and malignant glomus tumors: analysis of 52 cases, with a proposal for the reclassification of glomus tumors. Am J Surg Pathol.

[12] Standards of Practice Committee, Faulx AL, Kothari S (2017). The role of endoscopy in subepithelial lesions of the GI tract. Gastrointest Endosc.

[13] Vassiliou I, Tympa A, Theodosopoulos T, Dafnios N, Fragulidis G, Koureas A, Kairi E (2010). Gastric glomus tumor: a case report. World J Surg Oncol.

[14] Kang G, Park HJ, Kim JY (2012). Glomus tumor of the stomach: a clinicopathologic analysis of 10 cases and review of the literature. Gut Liver.

[15] Debol SM, Stanley MW, Mallery S, Sawinski E, Bardales RH (2003). Glomus tumor of the stomach: cytologic diagnosis by endoscopic ultrasound-guided fine-needle aspiration. Diagn Cytopathol.

